# Early-onset cerebellar ataxia in a patient with CMT2A2

**DOI:** 10.1101/mcs.a005108

**Published:** 2020-06

**Authors:** Ricardo Madrid, Sara R. Guariglia, Andrea Haworth, William Korosh, Maureen Gavin, Gholson J. Lyon

**Affiliations:** 1 Jervis Clinic, NYS Institute for Basic Research in Developmental Disabilities (IBR), Staten Island, New York 10314, USA;; 2 Congenica Ltd, Biodata Innovation Centre, Wellcome Genome Campus, Hinxton, Cambridge CB10 1SA, United Kingdom

**Keywords:** progressive cerebellar ataxia, severe global developmental delay

## Abstract

A 9-yr 8-mo-old right-handed female presented with a history of gait difficulties, which first became apparent at age 9 mo of age, along with slurred speech and hand tremors while holding a tray. Her past medical history was significant for global developmental delay, and she was attending fourth grade special education classes. On examination, she had an ataxic gait, dysarthria, absent deep tendon reflexes, and flexor plantar responses. There were no signs of optic atrophy or hearing loss. Nerve conduction studies were consistent with an axonal neuropathy. A fascicular sural nerve biopsy showed a marked decrease of myelinated fibers larger than 6 µm in diameter as compared with an age-matched control. By electron microscopy, clusters of degenerating axonal mitochondria in both myelinated and unmyelinated fibers were frequently found. Whole-exome sequencing revealed a heterozygous c.314C > T (p.Thr105Met) missense variant in *MFN2* in the patient but not in her mother. The father was unavailable for testing. The phenotypes with *MFN2* variants can be quite variable, including intellectual disability, optic atrophy, auditory impairment, spinal atrophy with or without hydromyelia, and hydrocephalus. We report here that early onset ataxia with intellectual disability can also be associated with *MFN2*-related Charcot–Marie–Tooth, Type 2A2A diagnosis, the most common type of autosomal dominant axonal neuropathy.

## CASE PRESENTATION

This 9-yr 8-mo-old right-handed female at the time of initial evaluation presented with a history of gait disturbance (HP:0001288) with frequent daily falls and inability to walk or sit straight since at least 9 mo of age, which prompted the start of physical therapy at that time. She also had slurred speech-impaired language (HP:0000750) and hand tremors (HP:0002378) while holding a tray. She would frequently drop things. She was attending fourth grade special education classes. Her phenotypic features, and those of her parents (when available), are listed in Table 1.

**Table 1.** Phenotypic features for CMT2A with early onset of ataxia

HPO code	HPO Term	Proband	Mother	Father	Sibling
HP:0001263	Global developmental delay	Y	N	N/A	N
HP:0001288	Gait disturbances	Y	N	N	N
HP:0001270	Motor delays	Y	N	N/A	N
HP:0000750	Delayed speech and language	Y	N	N/A	N
HP:0006957	Hypotonia	Y	N	N/A	N
HP:0002378	Hand tremors	Y	N	N/A	N
HP:0001251	Ataxia	Y	N	N	N
HP:0001260	Dysarthria	Y	N	N/A	N
HP:0002403	Positive Romberg sign	Y	N	N/A	N
HP:0002174	Postural tremor	Y	N	N/A	N

(HPO) Human Phenotype Ontology, (N/A) not available.

### Pregnancy and Perinatal

There was an unremarkable pregnancy and full-term vaginal delivery of an infant girl weighing 3.26 kg (7 lbs. 3 ounces) (38th percentile). The baby was breastfed for 1 mo, followed by bottle feeding.

### Development

The proband has a medical history significant for global developmental delay (HP:0001263) and progressive cerebellar ataxia (HP:0002073). The mother noted the infant could not sit independently at 9 mo of age, which prompted an evaluation by an early intervention specialist. She walked independently at 24 mo of age while receiving physical therapy.

### Physical Examination

On examination, the proband had gait ataxia (HP:0002066), dysarthria (HP:0001260), generalized hypotonia (HP: 0001290), absent deep tendon reflexes (HP:0001284), postural tremor (HP:0002174), and flexor plantar responses. Her Romberg sign was positive (HP:0002403), with eyes open and closed (see Table 1). There was a slight atrophy of the legs without foot deformity. There were no signs of optic atrophy, nystagmus, or hearing loss. The proband's presentation was initially suggestive of Friedreich's ataxia, but molecular analysis was negative. On her latest follow-up visit at age 12, she continued having frequent falls and, on examination, ataxic dysarthria and a very unsteady gait were again noted. Attempts at tandem gait were met with loss of balance, and the Romberg test was positive with eyes open and closed. There was persistent dysmetria in the finger to nose test and in the Archimedes spiral test.

### Previous Metabolic, Genetic, and Quantitative Testing

Workup done prior to our evaluation included serum amino acids, urine organic acids, urine copper, serum ceruloplasmin, creatine kinase, and aldolase, all of which yielded normal results.

**Figure 1.**
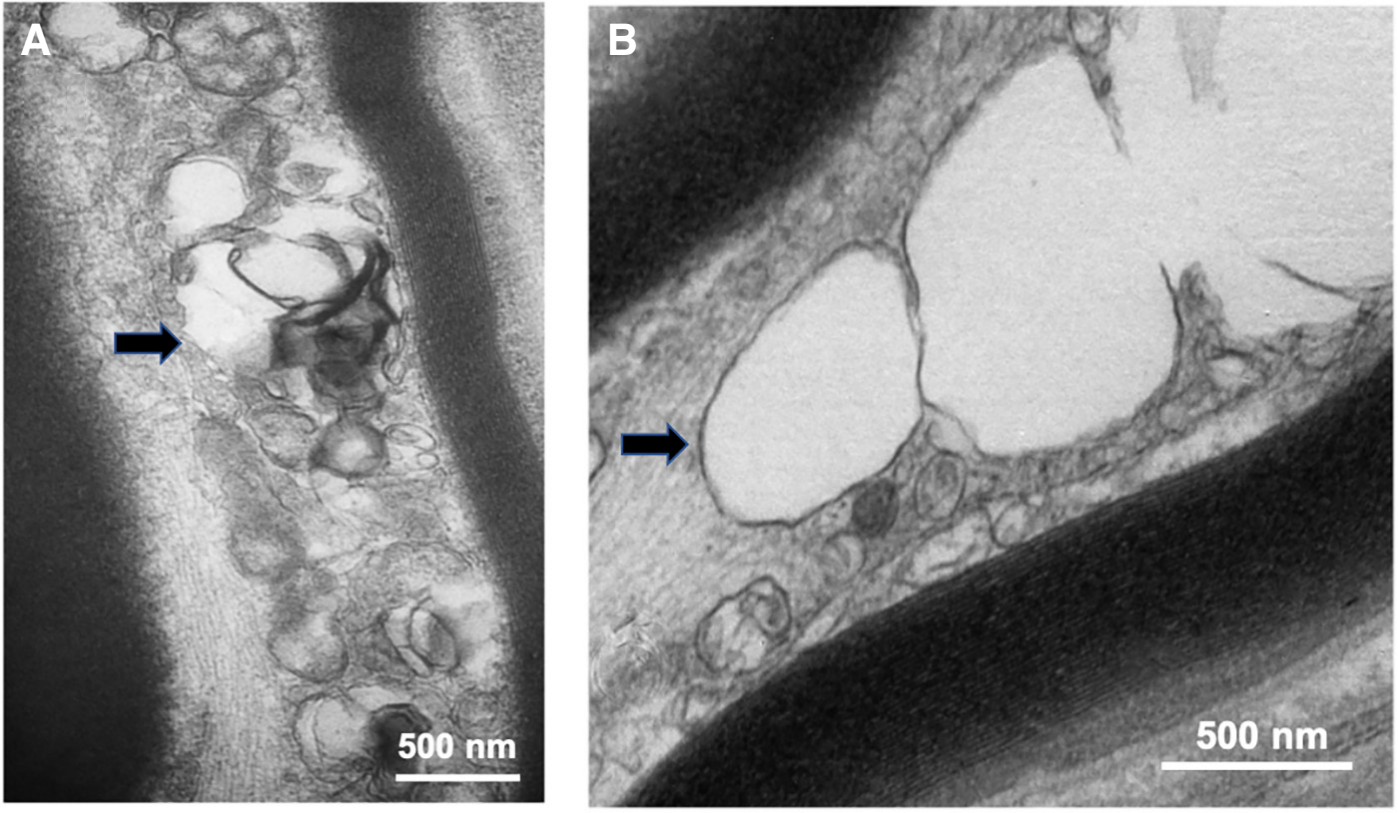
Electron microscopy studies. (*A*) At the ultrastructural level, clusters of normal and degenerating axonal mitochondria were frequently found in longitudinal sections of both myelinated and unmyelinated fibers (shown by black arrow). (*B*) Electron microscopy of mitochondria showing swelling and lack of organized cristae (shown by black arrow). The double membranes confirm the mitochondrial nature of these organelles.

The proband had a normal female karyotype, and the whole-genome chromosome single-nucleotide polymorphism (SNP) microarray analysis was also normal. Sensory nerve action potentials were normal except for the median nerve, whereas the compound motor action potentials and motor nerve conduction velocities were all in the low normal range. With the mother's informed consent, a right sural nerve biopsy was done with the patient under conscious sedation (at the age of 9 yr 8 mo old). The sural nerve was macroscopically thinner than normal. It was processed and embedded for light and electron microscopy by standard methods. Quantitative studies done as reported previously (Madrid et al. 2013) showed a transverse fascicular area less than half of an age-matched control (0.23 mm^2^ vs. 0.53 mm^2^) (Jacobs and Love 1985). The myelinated fiber density was also decreased mainly because of a loss of large myelinated fibers as compared with an age-matched control (7818 vs. 13,000/mm^2^) (Fig. 1A). At the ultrastructural level, clusters of normal and degenerating axonal mitochondria were seen in longitudinal sections of myelinated and unmyelinated fibers and, occasionally, in the cytoplasm of Schwann cells. Some mitochondria showed blown-out inner membranes, whereas others had no organized cristae (Fig. 1B).

### Family Background

There is no family history of similarly affected family members. The family is from the Dominican Republic, and there is no known consanguinity. The proband has one older brother with no developmental issue who attends mainstream high school.

## TECHNICAL ANALYSIS

Clinical exome sequencing was performed by Ambry Genetics, using their proprietary pipeline (see Supplementary Table 1 for exome sequencing statistics). This revealed a heterozygous NM_001127660.1: c.314C > T (p.Thr105Met) pathogenic variant in *MFN2* in the patient but not in her mother. The father and older brother were unavailable for testing. See Table 2 for complete exome findings.

**Table 2.** Exome findings

Gene	Genomic location	HGVS cDNA	HGVS protein	Zygosity	Parent of origin	Variant interpretation
*MFN2* gene (OMIM 608507)	Chromosome 1p36.2 NM_001127660.1:	NM_014874.3:c.314C > T	NP_055689.1: p.Thr105Met	Heterozygous	Not in the mother, the father was unavailable for testing	Pathogenic

(HGVS) Human Genome Variation Society.

Additional variant calling and interpretation of the Ambry whole-exome sequencing (WES) data was conducted by the U.K. state registered Clinical Scientists in Congenica software (Congenica Limited). This included repeat variant calling and filtering, with FASTQ data aligned to GRCh37 using BWA-MEM (0.7.12) and variant calling performed using GATK HaplotypeCaller (version 3.4-46). QC of fastq files was done with FASTQC (0.11.5) and VCF variant-based QC done using VariantRecalibrator (GATK). Variant annotation against all RefSeq and Ensembl transcripts was done via Ensembl Variant Effect Predictor (VEP; version 81). Variants were prioritized/ranked using Exomiser's Gene Pheno Score (version 10). Rare variants were identified by filtering using a maximum allele frequency setting of 0.5% in the following population data sets: ExAC, Exome Sequencing project (ESP), UK10K, and 1000 Genomes. Variant consequence filters were used to select variants predicted to impact coding sequence, consensus splice sites, and splice regions associated with RefSeq and Ensembl transcripts. Resulting variants were analyzed using Congenica software (Congenica Limited) using a gene-agnostic approach utilizing filtering by inheritance and phenotype driven variant prioritization (Smedley et al. 2015). A virtual gene panel approach was used for review of variants in 1201 genes known to be associated with ataxia. This analysis did not find any additional pathogenic variants.

Repeat Expansion Analysis was performed, with no positive findings, for the following 15 genes: *ATN1*, *ATXN1*, *ATXN10*, *ATXN2*, *ATXN3*, *ATXNl*, *ATXNB*, *ATXNBOS*, *BEAN1*, *CACNA1A*, *FMR1*, *FXN*, *NOP56*, *PPP2R28*, and *TBP*. For the repeat expansion analysis, blood or genomic DNA extracted from the provided specimen was fragmented and made into a library by ligating barcoded universal oligonucleotide adapters using a polymerase chain reaction (PCR)-free protocol. This prepared library was then sequenced by next-generation sequencing technology. Following alignment to the human genome reference (hg19/GRCh37), Illumina Expansion Hunter (v3) (Dolzhenko et al. 2017) was used to screen for abnormalities in short-tandem repeat (STR) length at specific loci within each gene tested. Any genes screening positive would have been validated using a gene-specific single-gene repeat-primed PCR test.

## VARIANT INTERPRETATION

The T105M variant found in this patient is not present in gnomAD v.2.1.1. This sequence change replaces threonine with methionine at codon 105 of the MFN2 protein (p.Thr105Met). The threonine residue is highly conserved and there is a moderate physicochemical difference between threonine and methionine. Computational analysis using SIFT and PolyPhen categorize this variant as likely pathogenic/deleterious. This variant has been shown to segregate with Charcot–Marie–Tooth disease type 2 (CMT2) in two families (Lawson et al. 2005; Wang et al. 2016) and has been reported in at least six unrelated individuals affected with CMT2 (Züchner et al. 2004; Kijima et al. 2005; Lawson et al. 2005; Amiott et al. 2008; Feely et al. 2011; Bombelli et al. 2014). This variant has been shown to occur de novo in at least two families (Chung et al. 2006; Xie et al. 2016). ClinVar contains an entry for this variant (Variation ID: 214652). Functional studies indicate that this variant causes defects in MFN2-mediated mitochondrial fusion in a dose-dependent manner (Detmer and Chan 2007; Amiott et al. 2008, 2009; Detmer et al. 2008). In addition, this missense change is located within a functionally conserved GTPase domain (amino acids 103–110) of the MFN2 protein (Lawson et al. 2005; Detmer et al. 2008; Calvo et al. 2009), and a significant number of previously reported MFN2 mutations have been found in this domain (Detmer et al. 2008). Based on these observations, we interpret this variant to be Pathogenic, according to the American College of Medical Genetics and Genomics (ACMG) guidelines (Richards et al. 2015), including (1) PS2, de novo (both maternity and paternity confirmed) in two prior individuals with the disease and no family history; (2) PS4, the prevalence of the variant in affected individuals is significantly increased compared with the prevalence in controls; (3) PS3, well-established in vitro or in vivo functional studies supportive of a damaging effect on the gene or gene product (performed in a research setting, so at reduced strength); (4) PM1, located in a mutational hotspot and/or critical and well-established functional domain (e.g., active site of an enzyme) without benign variation; (5) PM2, absent from controls; (6) PP1, cosegregation with disease in multiple affected family members in a gene definitively known to cause the disease; and (7) PP3, multiple lines of computational evidence support a deleterious effect on the gene or gene product.

Reanalysis of the WES findings by filtering for genes involved in cases of early-onset ataxia (Westra et al. 2019) did not identify any other variants. We also tested for CAG repeat expansions in the coding region of 15 genes known to be associated with spinocerebellar ataxia (SCA), but these results were negative, and furthermore, most SCAs are accompanied by significant cerebellar atrophy, which was not detected in the magnetic resonance imaging (MRI) of the brain in our patient. A limitation of our study is that it is formally possible that we missed repeat expansions in genes that are not yet formally associated with ataxia or that the sensitivity and specificity of the algorithm Illumina Expansion Hunter (v3) (Dolzhenko et al. 2017) was insufficient to detect repeat expansions in the 15 genes tested, despite the published data illustrating high accuracy.

## SUMMARY

Variants in *MFN2* cause CMT2A2, the most common type of autosomal dominant axonal neuropathy (Züchner et al. 2004). Most variants locate within or close to the GTPase G1 domain or within the coiled-coil domains. Most patients have an early onset and severe neuropathy, whereas later-onset cases have a milder disease course (Verhoeven et al. 2006). In some families as many as 25% of individuals carrying the variant may be asymptomatic with normal nerve conduction, although a detailed neuromuscular examination may suggest the trait (Lawson et al. 2005). The sural nerve biopsies of children with CMT2A2 show a marked decrease in large myelinated fibers and clusters of small and degenerating mitochondria accumulating primarily at the axon periphery of both myelinated and unmyelinated fibers (Vallat et al. 2008). Additional phenotypes found in patients with MFN2 variants include intellectual disability, optic atrophy (HMSN6), vocal cord palsy, spinal atrophy with or without hydromyelia, and hydrocephalus, all of which support the presence of central nervous system (CNS) involvement in CMT2A2 (Bombelli et al. 2014). *MFN2* (OMIM 608507) is localized to Chromosome 1p36.2 and it codes for the MFN2 protein that resides in the mitochondrial outer membrane. The protein contains an amino-terminal GTPase (guanosyltriphosphatase) domain and a transmembrane domain near the carboxyl terminus. The GTPase domain mediates mitochondrial fusion, whereas the carboxy-terminal coiled-coil domain mediates mitochondrial clustering. The MFN2 protein tissue specificity is ubiquitous and expressed at low level except in heart and kidney.

A heterozygous missense variant is reported here in a young girl with early-onset cerebellar ataxia. This early-onset cerebellar ataxia represents an expanded phenotype of the *MFN2* pathogenic T105M mutation. *MFN2* variants were also reported in individuals with clinical diagnosis of hereditary motor and sensory neuropathy (OMIM#601152) to which the clinical findings of this individual (positive Romberg sign, hyporeflexia) also fit, and in which the child is not yet old enough to have manifested possible optic atrophy. This individual might have a clinical course of both CMT2A and CMT6A, which is consistent with the clinical variability reported (Del Bo et al. 2008), although the patient's Romberg sign was positive with eyes open and closed, which is what would be expected if the sign is positive as a result of cerebellar involvement. In contrast, patients with CMT6 have a positive Romberg sign only with eyes closed, because in them the positivity is due to loss of position sense, which is compensated when the eyes are open. Furthermore our patient had ataxic dysarthria, which is not present in CMT6A. We performed a sural nerve biopsy that confirmed the presence of the mitochondrial abnormalities seen in patients with CMT2A2. It has been suggested (Chan 2007) that peripheral nerves may be specifically affected in CMT2A2 because they express low levels of *MFN1* and rely primarily on *MFN2* for mitochondrial fusion. Abnormal mitochondrial trafficking has been also implicated in the selective susceptibility of the longest peripheral axons to *MFN2* mutations (Baloh et al. 2007). Purkinje cells express predominantly *MFN2*, as shown in conditional Mfn2 knockout mice; thus, *MFN1* cannot protect from the mitochondrial fusion defects caused by *MFN2* mutations (Chen et al. 2007). The involvement of the cerebellum in CMT2A2 patients, as presented here, may be more frequent than is currently acknowledged, and mutations in *MFN2* should be considered among genes possibly associated with not only infantile-onset cerebellar ataxias but also pathologically small sural nerves, which up to now have been reported in spinocerebellar ataxia type 2 and in cerebellar ataxia, neuronopathy, and vestibular areflexia syndrome (CANVAS).

## ADDITIONAL INFORMATION

### Database Deposition and Access

The exome sequencing data were generated as part of clinical testing, and the raw data were obtained from Ambry Genetics for re-interpretation. This does not include consent for sharing the data to public databases. The variant has been submitted to ClinVar (https://www.ncbi.nls.nih.gov/clinvar) and can be found under accession number VCV000214652.3.

### Ethics Statement

Both oral and written patient consent were obtained for research and publication, with approval of protocol #7659 for the George A. Jervis Clinic by the New York State Psychiatric Institute–Columbia University Department of Psychiatry Institutional Review Board. Family consent was not given for photography of the subject.

### Acknowledgments

Initial clinical variant interpretation was performed by Dima El-Khechen at Ambry Genetics**.** Appreciation is extended to the family. Elaine Marchi provided editorial assistance. The authors thank the Genome Aggregation Database (gnomAD) and the groups that provided exome and genome variant data to this resource. A full list of contributing groups can be found at http://gnomad.broadinstitute.org/about.

### Author Contributions

R.M. performed clinical evaluation, designed the experiment, and wrote the manuscript; S.R.G. set up and aligned the electron microscope and helped with the micrographs; A.H. performed clinical exome re-interpretation; W.K. performed clinical evaluation; M.G. assisted with repeat expansion testing; and G.J.L. performed whole-exome sequencing analysis and edited the manuscript.

### Funding

Funding for this report was provided by the George A. Jervis Clinic of the New York State Institute for Basic Research in Developmental Disabilities (IBR), New York State Office for People with Developmental Disabilities and the Department of Human Genetics, Laboratory of Genomic Medicine internal budget.

### Competing Interest Statement

The authors have declared no competing interest.

## Supplementary Material

Supplemental Material
