# Supplementary material for: Early-onset cerebellar ataxia in a patient with CMT2A2
Source: Cold Spring Harb Mol Case Stud. 2020 Jun;6(3):a005108. doi: 10.1101/mcs.a005108 (PMC7304361; doi:10.1101/mcs.a005108)
Supplement: Supplemental Material [file supp_6_3_a005108__index.html]

Supplemental Material 

# Early-onset cerebellar ataxia in a patient with CMT2A2

## Supplemental Material

- Supplementary\_Table\_1.docx
