# Supplementary material for: Early-onset cerebellar ataxia in a patient with CMT2A2
Source: Cold Spring Harb Mol Case Stud. 2020 Jun;6(3):a005108. doi: 10.1101/mcs.a005108 (PMC7304361; doi:10.1101/mcs.a005108)
Supplement: Supplemental Material [file supp_mcs.a005108_Supplementary_Table_1.docx]

**Supplementary Table 1 - Exome sequencing statistics**

|  | **Depth of coverage** | |
| --- | --- | --- |
| **Relationship** | % bases >10x | % bases > 20x |
| Proband | 98.74 | 98.22 |
| Mother | 95.79 | 81.02 |
| Average | 97.27 | 89.62 |
